# The Radical Treatment of Stricture of the Rectum Viewed from the Standpoint of the Etiology

**Published:** 1894-02

**Authors:** John P. Bryson

**Affiliations:** St. Louis, Mo.


### THE RADICAL TREATMENT OF STRICTURE OF THE RECTUM VIEWED FROM THE STANDPOINT OF ITS ETIOLOGY.

BY JOHN P. BRYSON, M. D., ST. LOUIS, MO.

[Written for Mathews' Medical Quarterly.]

Stricture of any hollow organ is classed as an obstructive disease, is described as an obstruction to the flow of physiological currents, is defined as an obstacle to the passage of an instrument, is diagnosticated as a mechanical narrowing, and until very recently at least, and even now by the great majority of surgeons, is treated as a purely mechanical affair and after a purely mechanical fashion. One recent writer has even gone so far as to propose a purely mechanical etiology for the disease, viz., friction. One would think that the study of the subject of stricture involved only physical problems, dealt with questions of non-living matter, and that biological laws were of no, or at most of very little, importance. And yet the obstructive, that is to say the mechanical element in the pathological entity we know as stricture, is as purely a symptom as dropsy is a symptom of certain cardiac, renal or hepatic diseases.

It is to be said in favor of the "physical" school that they were logical in their ways, even to the extremity of treatment. The mechanical classification, definition and conception was, logically enough, followed by mechanical therapeutics. They found what was thought to be a mechanical obstacle, and they attacked it mechanically. Much perseverance has been shown in this method. Almost invariably the obstacle returned shortly to plague them, but it was again and again attacked and in the same way.

Yet, both teleologically and etiologically, the subject of stricture is well enough developed on the pathological side to justify many positive and most valuable conclusions respecting definition, classification and method of radical treatment. Viewed from this standpoint, the stricture tissue is built up, not with the purpose of obstructing a duct, but in order to resist the return of the contents of that duct into the body from which it has perhaps but recently been excreted, or the entrance by some other channel than the proper and physiological one. Thus, in the case of the urethra—where the process has been most carefully studied—absence of, or a certain amount of damage to, a zone of mucous membrane is followed by the growth of stricture tissue around that part of the duct. It is to be noted that this stricture-building does not begin until the overlying mucous membrane ceases to serve as an efficient physiological barrier to urine leakage; but that it not only ceases to develop, but disappears by absorption as soon as the physiological (urine-tight) lining of the duct is restored. Observation of these facts led Reginald Harrison to formulate the law that urethral stricture-building was the result of urine leakage. That the purpose of the stricture growth is not primarily to close or obstruct the duct, is to be seen in the fact that the same growth takes place in other parts of the body, if urine is kept a long time in contact with them when denuded of their physiological protection, for instance, the walls of fistulæ. These fistulous walls are histologically identical with the stricture bands, and undergo involution so soon as the urine passes by the natural channels and no longer remains in contact with them. It thus becomes possible to formulate the proposition that urethral stricture has its beginning in the urethral mucosa, and to say that without damage or destruction of the mucous membrane there would be no stricture-building.

If such a urethral stricture be cut (externally or internally), violently stretched and torn (divulsed), or caused to inflame and disappear by absorption (gradual dilatation), it will quickly return unless prevented by the persistent introduction of dilating instruments. Thus mechanical treatment deals only with the mechanical symptom, and is palliative, not radical. In other words, we do not cure a disease by abolishing one of its symptoms.

If, however, we exsect the strictured area and accurately suture the healthy mucous membrane behind to the healthy mucous membrane in front, getting union *per primam;* or, if we do a urethrectomy for the strictured portion and transplant some healthy mucous membrane, the lumen of the duct remains uncontracting without the necessity of reintroducing dilating instruments. In the one case we have restored the mucous lining of the duct in its integrity, in the other we have not.

*Mutatis mutandis*, what has been stated of urethral will apply with equal force to rectal strictures. In a paper read by me at the Newport meeting of the American Association of Genito-Urinary Surgeons (1889),* I offered as a pathological definition of urethral stricture, chronic, contracting peri-urethritis (*Urethritis stringens chronica*). Holding close to this purely pathological definition, it is not difficult to distinguish between stricture and those more mechanical coarctations with which it is so frequently confounded. The one most nearly resembling stricture is that form of congenital stenosis which assumes a form of a diaphragm, and is usually found in the lower third of the rectum. On the mechanical definition there is no adequate justification in the exclusion of this from the classification of stricture. Anatomically it is a linear stricture of the most typical kind; but, measured by the pathological definition, it falls entirely out of the category. Let us apply the measure.

There are certain features that are common to stricture of all hollow organs. We will apply the measure as they are enumerated: (1) In stricture there is always a damaged and inefficient mucous membrane or none at all (cicatricial stricture) lining the affected portion; in congenital stenosis the mucosa is intact. (2) In stricture there is a chronic inflammation which constantly adds new tissue to the stricture mass, persistently pushing the remainder of mucous membrane toward the axis of the duct, and often forcing it up into longitudinal ridges; in congenital stenosis there is no inflammation of any kind, no increasing, narrowing, no folding up or pushing inward of the mucosa. (3) In stricture there is a pathological state, slow-moving but persistent throughout stricture-building; in congenital stenosis there is no pathological condition, but only an anatomical and stationary one. (4) Therapeutically the contrast is equally striking, for in stricture linear proctotomy does not cure radically, while in congenital stenosis linear division or even nicking and tearing bring permanent results.

*Journal of Cutaneous and Genito-Urinary Diseases, June, 1889.

Measured in the same manner and by the same standard, other diseases and conditions, such as periproctitic abscess and fibrous bands, peritonitic thickenings and contractions, pelvic tumors, prostatic overgrowths and prostatic swellings, yea, even the much-vaunted and apparently never-to-be-neglected muscle spasms, are excluded and cast into the extra-stricture world, let them pursue ever so loud an "obstructive" policy. Cancer, beginning as it does in the mucosa and possessing obstructive features of the highest respectability, cannot enter in under the definition of "chronic contracting periproctitis," but must remain plain cancer of the rectum.

To all of these conditions and diseases, however, belong obstructive symptoms of the most important kind, which demand surgical intervention and tax our surgical therapeutics, but are only symptoms and not essential pathological characters, even though many of them threaten or destroy life only by obstructing the duct.

It is clear, then, that stricture, under the definition given above, can only be radically cured by restoring the mucous membrane to its physiological integrity. It is the object of this paper to point out and to emphasize this point, and not to discuss the surgical means at our disposal for accomplishing this object. I shall reserve this matter for another communication soon to be made, and content myself with mentioning the means at present in sight, viz.: (*a*) proctectomy with circular suture of the divided (healthy) walls; (*b*) proctectomy with transplantation; (*c*) curetting with transplantation on the denuded base (perhaps after a colotomy); and (*d*) gradual, (inflammatory, atrophic) dilatation, followed by topical treatment intended to restore to an efficient condition the diseased epithelial lining.

